# Comparison of Threshold Saccadic Vector Optokinetic Perimetry (SVOP) and Standard Automated Perimetry (SAP) in Glaucoma. Part II: Patterns of Visual Field Loss and Acceptability

**DOI:** 10.1167/tvst.6.5.4

**Published:** 2017-09-06

**Authors:** Alice D. McTrusty, Lorraine A. Cameron, Antonios Perperidis, Harry M. Brash, Andrew J. Tatham, Pankaj K. Agarwal, Ian C. Murray, Brian W. Fleck, Robert A. Minns

**Affiliations:** 1 University of Edinburgh, Edinburgh, Scotland, United Kingdom; 2 Glasgow Caledonian University, Glasgow, Scotland, United Kingdom; 3 Heriot Watt University, Edinburgh, Scotland, United Kingdom; 4 Princess Alexandra Eye Pavilion, Edinburgh, Scotland, United Kingdom; 5 Royal Hospital for Sick Children, Edinburgh, Scotland, United Kingdom

**Keywords:** eye movement perimetry, saccadic eye movements, SVOP, visual field

## Abstract

**Purpose:**

We compared patterns of visual field loss detected by standard automated perimetry (SAP) to saccadic vector optokinetic perimetry (SVOP) and examined patient perceptions of each test.

**Methods:**

A cross-sectional study was done of 58 healthy subjects and 103 with glaucoma who were tested using SAP and two versions of SVOP (v1 and v2). Visual fields from both devices were categorized by masked graders as: 0, normal; 1, paracentral defect; 2, nasal step; 3, arcuate defect; 4, altitudinal; 5, biarcuate; and 6, end-stage field loss. SVOP and SAP classifications were cross-tabulated. Subjects completed a questionnaire on their opinions of each test.

**Results:**

We analyzed 142 (v1) and 111 (v2) SVOP and SAP test pairs. SVOP v2 had a sensitivity of 97.7% and specificity of 77.9% for identifying normal versus abnormal visual fields. SAP and SVOP v2 classifications showed complete agreement in 54% of glaucoma patients, with a further 23% disagreeing by one category. On repeat testing, 86% of SVOP v2 classifications agreed with the previous test, compared to 91% of SAP classifications; 71% of subjects preferred SVOP compared to 20% who preferred SAP.

**Conclusions:**

Eye-tracking perimetry can be used to obtain threshold visual field sensitivity values in patients with glaucoma and produce maps of visual field defects, with patterns exhibiting close agreement to SAP. Patients preferred eye-tracking perimetry compared to SAP.

**Translational relevance:**

This first report of threshold eye tracking perimetry shows good agreement with conventional automated perimetry and provides a benchmark for future iterations.

## Introduction

Perimetry is central to the management of patients with glaucoma, and is used to aid diagnosis, gauge disease severity, and detect and measure rates of progression. Automated threshold perimetry using a white stimulus on a white background (standard automated perimetry, SAP) has become the accepted gold standard. SAP involves measuring the differential light sensitivity (DLS) at several test locations, with algorithms used to vary stimulus brightness and determine threshold values. Defining thresholds using a full threshold test is time-consuming and onerous for the patient, which can lead to reduction in test reliability and increased visit-to-visit variability. Although testing times have been reduced significantly using Swedish Interactive Threshold Algorithm (SITA) strategies, the common perception of visual field testing from patients is that it is difficult to perform.

An investigation of patients' opinions of tests used in the management of glaucoma, found SAP to be the least popular commonly performed procedure.^1^ Similar findings have been reported recently using patient focus groups, with patients expressing the opinion that SAP was in need of modernization.^2^ In addition, patients undergoing SAP have been found to experience higher levels of anxiety than those undergoing other tests, such as retinal imaging, and those who were anxious before undergoing SAP produced poorer reliability test results.^3^ There is a need for a more acceptable visual field test for patients.

Pioneering work by Damato has shown that the visual field could be evaluated by moving the eye rather than the test stimulus (oculokinetic perimetry),^4^ but only recently have several groups explored the possiblity of performing perimetry using computerized eye tracking.^5–8^ Studies using eye tracking also have shown that assessing the speed and accuracy of saccadic eye movements also may be useful for detecting disease, with differences in saccades noted between healthy individuals and those with glaucoma.^9–12^ Eye tracking can be used to monitor patient gaze responses to peripheral stimuli, with the advantage that it does not require the patient to press a response button. Additionally, by continually monitoring the patient's eye location and adjusting the position and size of the stimuli accordingly, perimetry can be performed without the need for a chin rest. We previously described a method of eye tracking perimetry termed saccadic vector optokinetic perimetry (SVOP).^13–16^ SVOP initially was developed as a suprathreshold test for children who struggle to maintain fixation and produce the timely and reliable responses needed for SAP. We recently developed a threshold SVOP test and have shown that threshold visual field sensitivities obtained using this device are correlated closely with SAP threshold values.^17^ The ability to perform threshold visual field testing using an eye tracker has the potential to offer an alternative method of perimetry to those who struggle with conventional testing. In the present study we compared patterns of visual field loss detected by SAP and threshold SVOP and examined patient perceptions of each technique.

## Methods

This cross-sectional study included patients with glaucoma and healthy subjects. Patients with glaucoma were recruited from a nonconsecutive series of patients attending the glaucoma clinic at the Princess Alexandra Eye Pavilion, Edinburgh. Healthy subjects were required to have no previous history of glaucoma or visual field defect and no neurologic conditions that might affect the visual field. All subjects provided written informed consent. The study adhered to the tenets of the Declaration of Helsinki and was approved by the South-East Scotland Research Ethics Committee, NHS Lothian. Details of the inclusion and exclusion criteria have been described previously.^17^

All patients attending the glaucoma clinic underwent best-corrected visual acuity, slit lamp biomicroscopy, intraocular pressure measurement, pachymetry, gonioscopy and dilated funduscopy. The diagnosis of glaucoma was made by a glaucoma specialist, based on the presence of typical glaucomatous changes in optic disc morphology and a glaucomatous visual field defect on SAP using the Humphrey visual field analyzer (HFA) SITA Fast 24-2 test (Carl Zeiss Meditec, Inc., Dublin, CA). All subjects performed SAP and threshold SVOP in both eyes and a random sample of subjects underwent repeat SAP and SVOP testing in one eye during the same session with suitable breaks if required. Patients with strabismus or a history of eye movement disorders were excluded. All patients had a best corrected vision of better than 0.3 logMAR. In the healthy group, eyes with a visual acuity worse than 0.15 logMAR were excluded.

### SVOP Test

**Figure 1:**
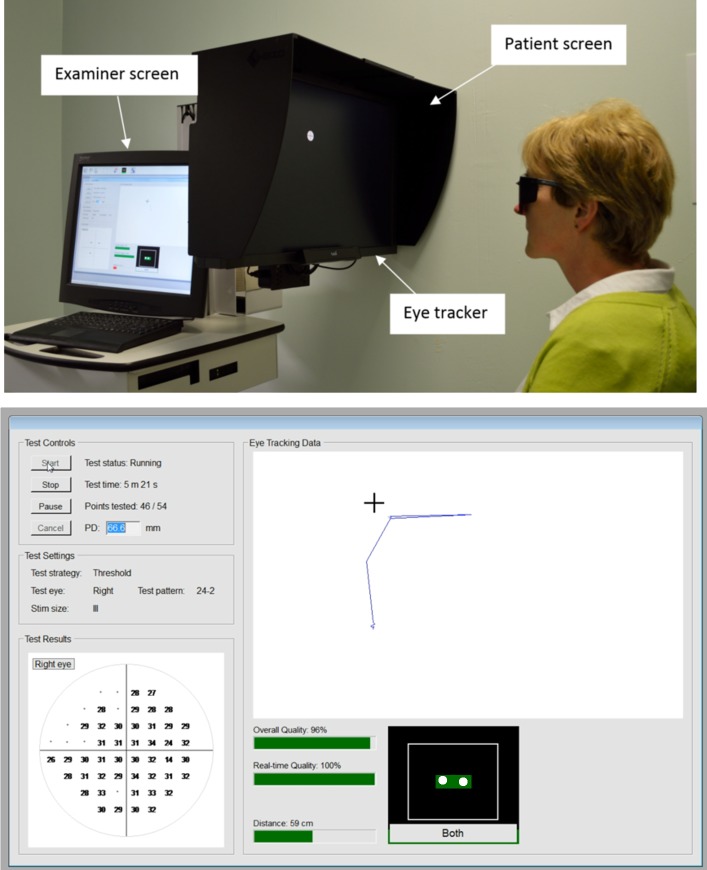
Photo of the threshold SVOP device during testing and screen capture of the examiner screen during testing.

The threshold SVOP device has been previously described.^17^ Threshold SVOP consists of a personal computer with an examiner screen, a 24″ high-resolution LCD patient screen (Eizo ColorEdge CG243W) and an eye tracker (IS-1 model; Tobii Technology, Stockholm, Sweden; Fig. 1).

Patients were seated in front of the screen with their eyes aligned with its center, at a testing distance of approximately 55 cm, measured using an eye tracking–based on-screen tool. Monocular testing was performed using custom made test spectacles allowing transferable, full aperture prescription lenses (55 mm diameter) if required, while also occluding the nontest eye with an infrared bandpass filter that enabled the eye tracker to detect the position of the occluded eye. The test commenced with the patient fixating on a central fixation stimulus. A test stimulus then was presented peripherally. During testing, the patient was instructed to follow their natural reaction to fixate towards any peripheral stimulus perceived. The eye tracker evaluated responses to the stimuli presented on the display screen, and custom software determined whether stimuli had been perceived based on the direction and amplitude of the subject's gaze response. The subject then was required to fixate on the peripheral stimulus until another was perceived. Stimuli were equivalent in size to Goldmann III and each stimulus was presented for 200 ms using coordinates equivalent to the SAP 24-2 test pattern. The size and position of the stimuli were automatically adjusted to compensate for changes in the patient's position during testing to ensure the perceived stimulus size remained constant.

The screen was calibrated using a Look-Up Table (LUT) pairing the grey-levels of each pixel to the corresponding required background (10 Cd/m^2^) and stimulus luminance levels. Stimuli luminance levels replicated the luminance values corresponding to 14 to 40 dB on the HFA. Thresholds were obtained using a 4-2 bracketing strategy and began by testing four “seed” locations (one in each quadrant), which then were used to set the starting stimulus luminance levels for neighboring locations, which in turn were used to calculate the remaining starting luminance levels. The SVOP threshold sensitivity values were matched in luminance to those of the HFA to allow direct comparison.

Before testing all subjects underwent a 20-second demonstration. SVOP testing then began with a calibration sequence, in which the subject was required to follow a stimulus to nine different screen locations to produce accurate eye gaze data during testing. Incomplete SVOP and unreliable SAP tests were excluded from analysis. An incomplete SVOP test was any test that was stopped prematurely, leaving any threshold values undetermined and an unreliable SAP test was defined as a test with a false-positive response rate exceeding 15%.

### Questionnaire

On completion of testing, subjects were asked to complete a short questionnaire regarding their experience and perceptions of SVOP and SAP. The questionnaire consisted of three questions regarding ease of test, perceived duration of testing, and test comfort, with responses recorded using a 5-point Likert scale. A final question asked patients to indicate their preferred test (SVOP or SAP).

### Modifications to SVOP During the Study

During the study, improvements were made to version 1 (v1) of the SVOP software and version 2 (v2) was developed. Modifications included: (1) an indicator of correct patient position, (2) an improved interactive demonstration test, (3) an increased time for determining fixation and a new fixation target with central pulsating crosshairs to improve fixation, and (4) modification of the starting luminance levels, calculated from “seed” location and neighboring location threshold levels to ensure they were never below 18 dB. This ensured that threshold point results would never be determined from a single stimulus decision. Software v2 also included two bug fixes, one relating to incorrect screen stimuli positions calculated under rare circumstances, and a second relating to incorrect calculation of the starting luminance level for one field point location. Subjects completing testing using v1 and v2 were asked to complete the questionnaire, however only results from software v2 were used for analysis of visual field defect patterns.

### Data Analysis

**Figure 2:**
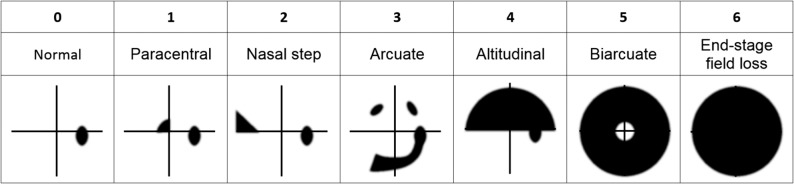
The patterns of visual field loss used for classification in the study. Adapted from the study of Broadway.^18^

To compare the patterns of visual field loss detected by threshold SVOP and SAP, visual field threshold sensitivity plots (greyscale) and sensitivity values were reviewed by a panel of three graders (one glaucoma specialist ophthalmologist and two research optometrists experienced in glaucoma) who reached a consensus on each visual field result. Grading was performed in two batches, each batch corresponding to a device, so that SVOP and SAP results from a single patient were not performed consecutively. Graders assigned visual fields into seven groups: 0, normal; 2, paracentral; 3, nasal step; 4, arcuate; 5, altitudinal; 6, biarcuate; and 7, end-stage field loss (Fig. 2).^18^ The purpose of this classification was not to represent the natural progression of glaucomatous field loss, but rather to allow broad stratification of different patterns of glaucomatous field loss. Graders were permitted to assign visual field results to multiple categories if more than one type of defect was felt to be present. For example, a plot with paracentral and arcuate loss could be classified as categories 1 and 3.

The agreement between SVOP and SAP classifications, using the highest number category if more than one was assigned, was compared qualitatively by cross-tabulation. Sensitivity and specificity of SVOP (v1 and v2) were calculated by comparison of normal (category 0) and abnormal (all other categories) results using SAP as the reference standard. The agreement between SVOP v1 and v2 and SAP also was assessed qualitatively by cross-tabulation. A random subset of patients underwent repeat SAP and SVOP testing to determine whether the patterns of visual field loss detected using each device were repeatable. Repeated results were cross-tabulated and performance assessed using descriptive statistics.

For group comparison tests, normality assumption was assessed by inspection of histograms and using Shapiro-Wilk tests. For comparison of two groups, Student's *t*-tests were used for normally distributed variables and the Wilcoxon rank-sum test for continuous nonnormal variables. For comparison of more than two groups, 1-way analysis of variance (ANOVA) was used. All tests were 2-sided and a *P* value less than 0.05 was considered statistically significant. Statistical analyses were performed with SPSS version 21.

## Results

The study included 59 healthy subjects and 103 with glaucoma. Healthy subjects were younger than those with glaucoma (65.7 ± 5.8 vs. 70.6 ± 8.79 years, respectively; *P* < 0.001). Table 1 summarizes the number of SAP and SVOP tests by subject, including the number of tests excluded due to incomplete tests or poor reliability. A total of 99 subjects underwent 191 SVOP v1 tests and 63 underwent 124 SVOP v2 tests. Of the 191 SVOP v1 tests, 39 (20%) were excluded due to inability to complete the test, decreasing to 5 of 124 tests (4%) with improvements in the SVOP software (v2). Following exclusion of incomplete and poor reliability tests, 142 comparison pairs (equivalent SVOP and SAP tests) for SVOP v1 and 111 comparison pairs for SVOP v2 were available for analysis.

**Table 1:**
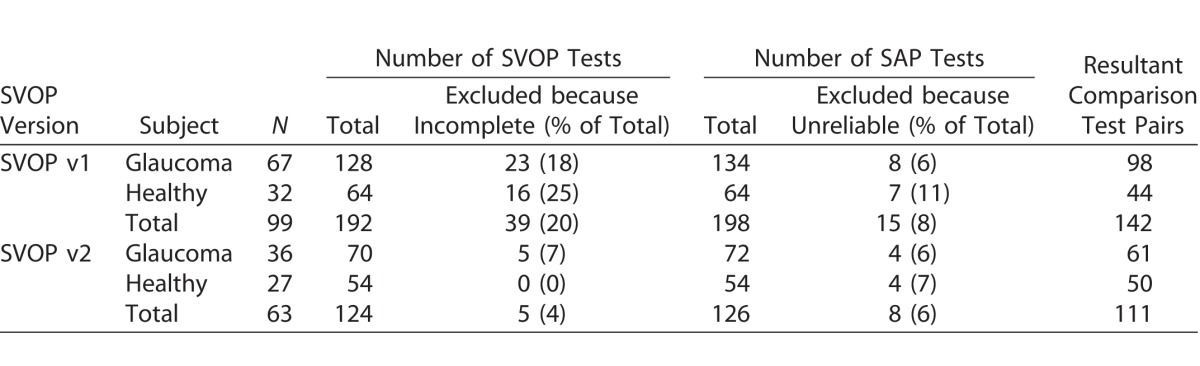
Number of Subjects and Tests (SVOP v1, v2 and SAP) Performed (Excluding Repeat Tests), the Number (and Reason) of Excluded Tests and the Resultant Comparison Test Pairs Used for Analysis

### Visual Field Defect Classification

Using the broad classification of normal (category 0) or abnormal (any other category), the sensitivity and specificity of SVOP were determined using SAP as the reference standard. The 2 × 2 contingency table for SVOP v2 is shown in Table 2, showing a sensitivity of 97.7% and specificity of 77.9% for SVOP v2 compared to SAP. SVOP v1 had a sensitivity of 95.3% and specificity of 61.0%.

**Table 2:**
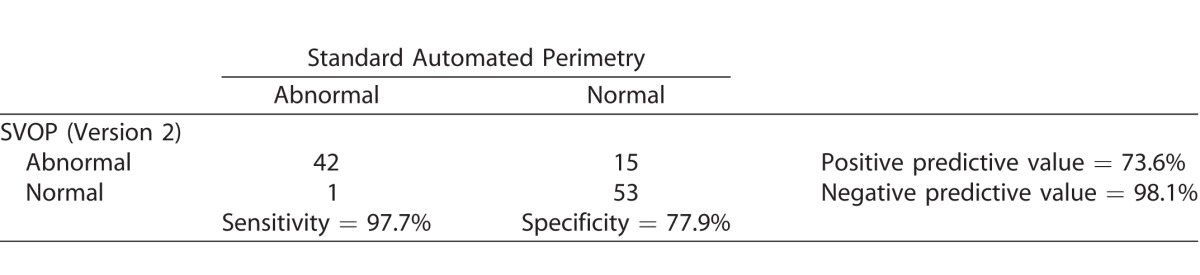
2 × 2 Contingency Table Comparing SVOP v2 with SAP

The detailed cross tabulation of the visual field defect categories between SVOP v2 and SAP is shown in Table 3 with figures in bold showing the number of cases where agreement occurred. A total of 61 test pairs (SAP and SVOP v2) were available in patients with glaucoma, with complete agreement in classification found in 33 (54%) and a further 14 (23%) disagreeing by one category. SVOP v1 had similar agreement, with complete agreement in 23 (69%) cases and disagreement by only one category in 14 (14%). The full cross-tabulation results for SVOP v1 are shown in the Supplementary Material. Supplementary Figure S1 shows several examples of threshold visual fields obtained from SAP and SVOP for patients included in the study.

**Table 3:**
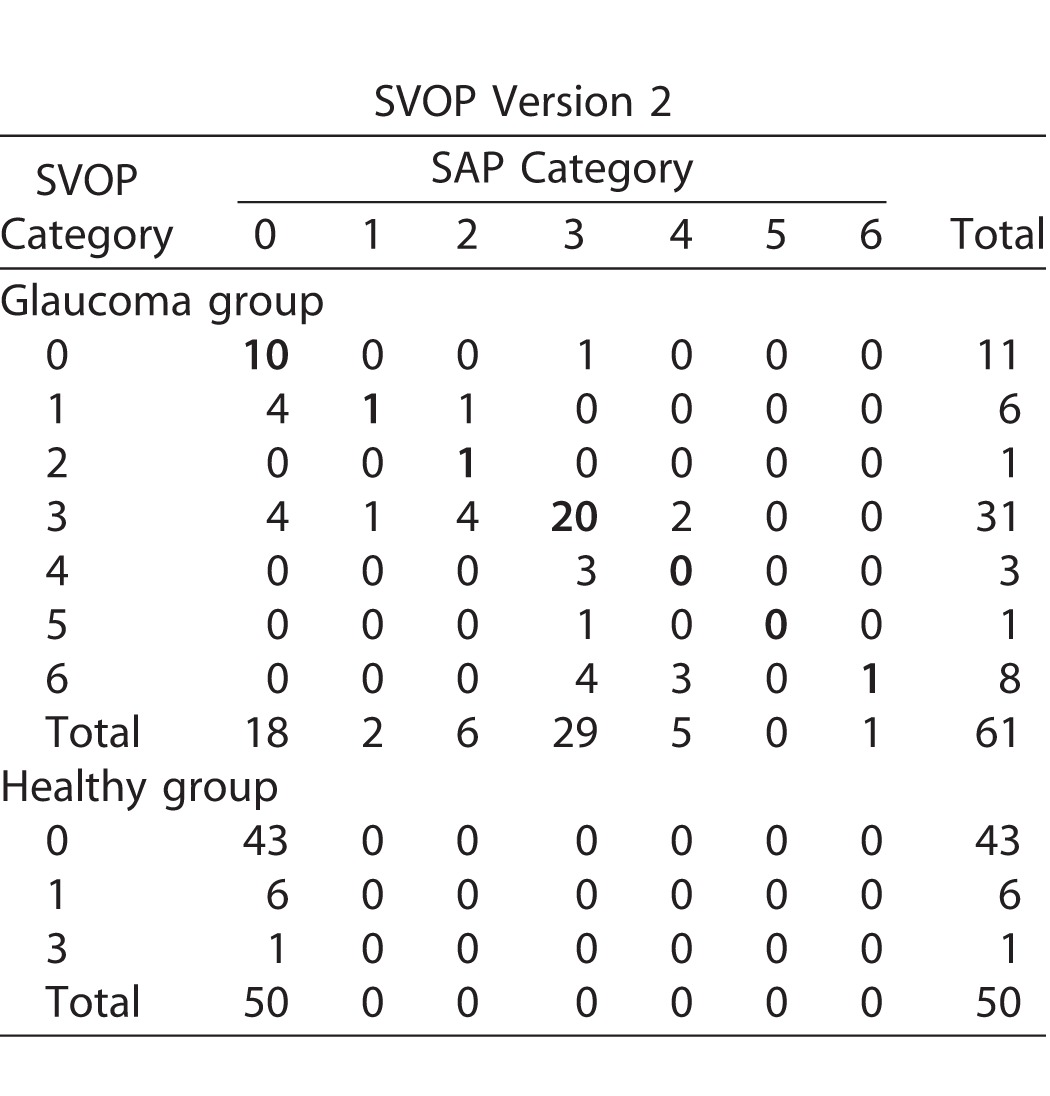
Comparison of Clinical Visual Field Categories for SAP and SVOP v2 in Healthy and Glaucomatous Subjects

### Repeatability of SVOP and SAP Pattern Classifications

Table 4 shows the number of subjects who underwent repeat SVOP and SAP testing, including the number of tests excluded due to incompleteness or poor reliability.

**Table 4:**
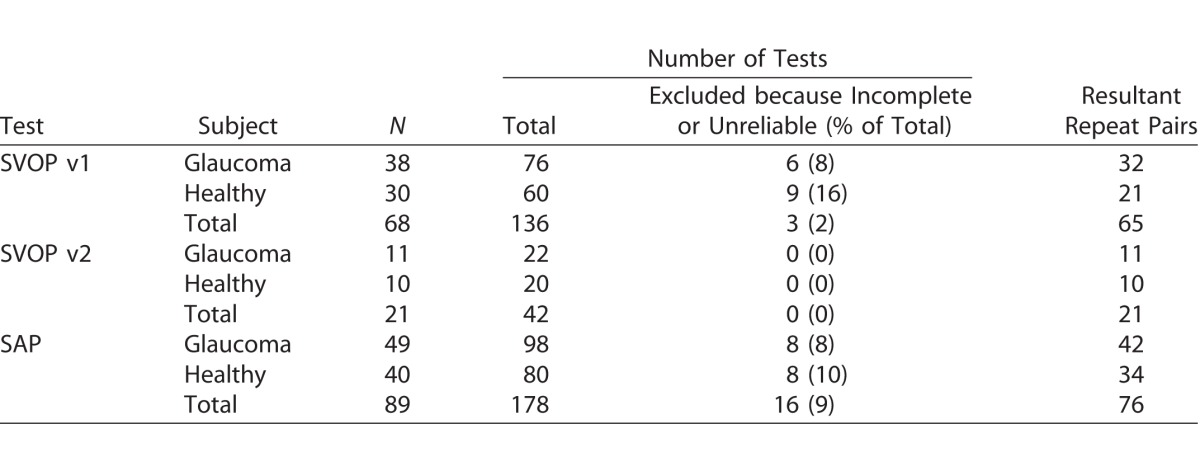
Number of Subjects Performing Repeat SVOP or SAP with Resulting Number of Repeat Pairs Used for Analysis of Repeatability of Identifying Patterns of Visual Field Loss

Repeat field tests were compared to the initial tests for each patient and the agreement in classification of pattern of field defect was compared (Table 5). SVOP v2 had similar reproducibility compared to SAP, with 86% and 91% showing complete agreement respectively.

**Table 5:**
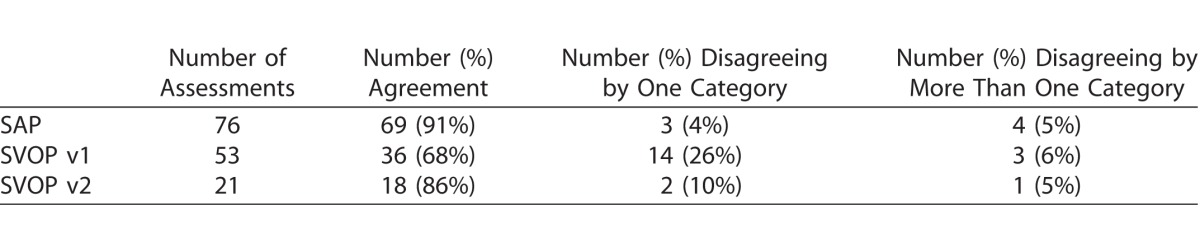
Agreement in Visual Field Defect Pattern Classification for Repeat Tests for SAP and SVOP

### Acceptability

**Figure 3:**
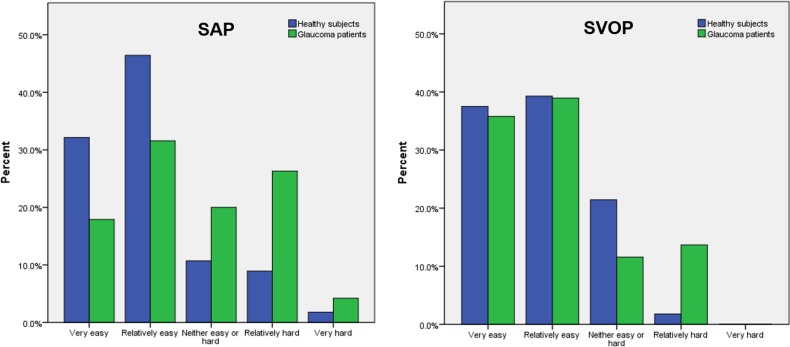
Participant perceived ease of performing SVOP (v1 and v2) and SAP.

**Figure 4:**
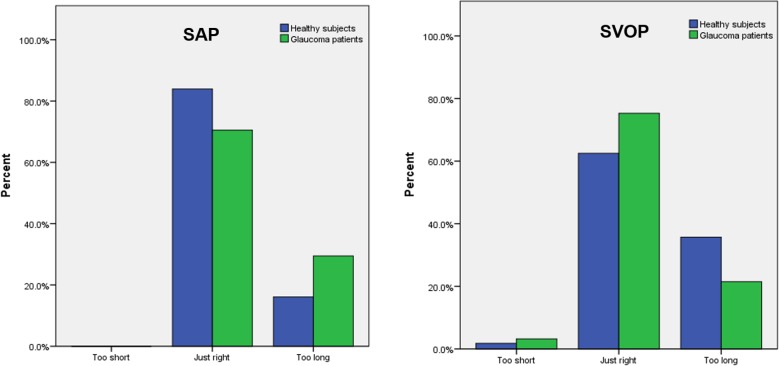
Participant perceived test duration for SVOP and SAP.

**Figure 5:**
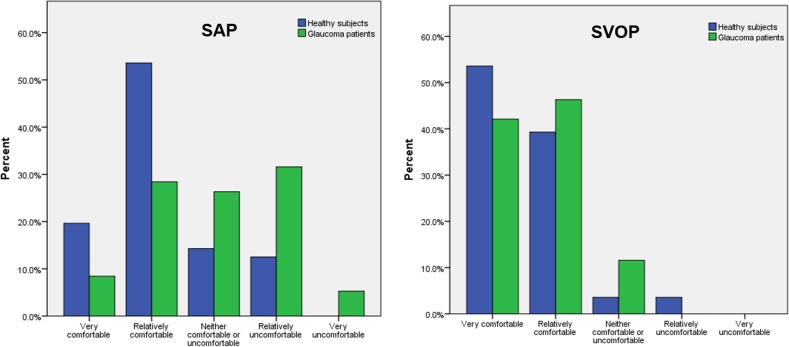
How comfortable the subject felt while performing SAP or SVOP testing.

**Figure 6:**
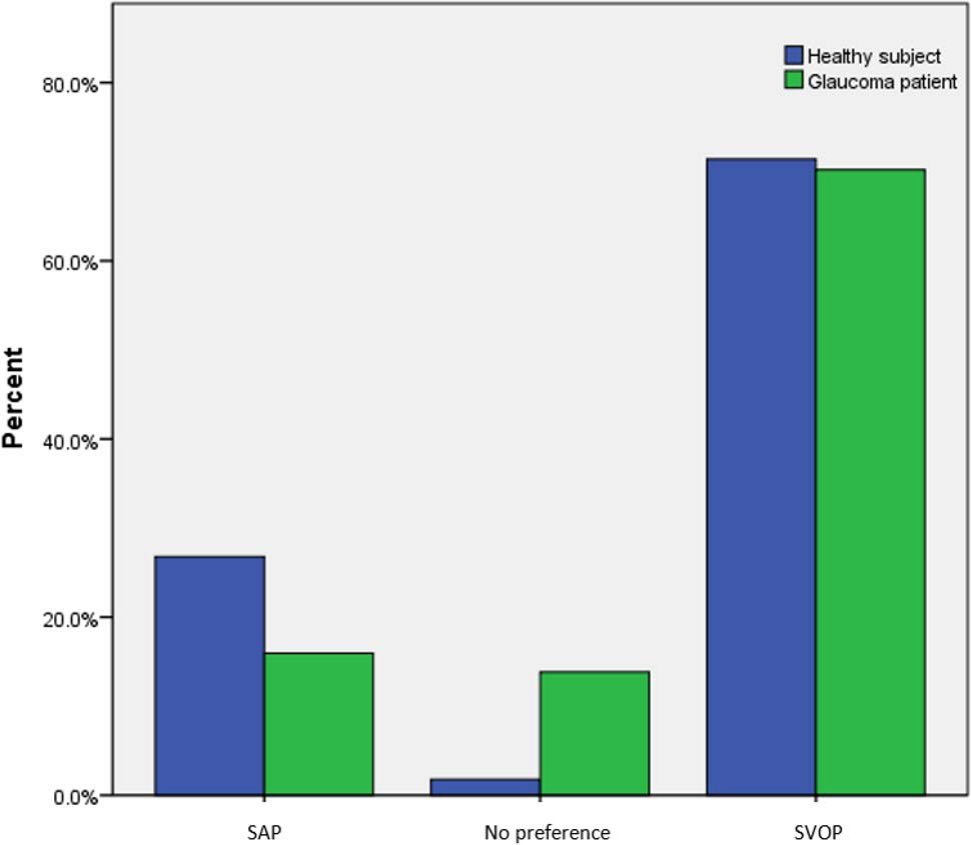
Participant preference regarding test type (SAP or SVOP).

The results of the patient questionnaire examining test preferences are shown in Figures 3 to 6. A greater proportion of participants, and particularly those with glaucoma, reported SAP to be hard or relatively hard to perform compared to SVOP (v1 and v2). Although SVOP took longer than SAP, most patients thought the test time was “just right.” Figure 5 shows the patients' opinion on how comfortable they were during testing. Of the patients and healthy subjects, 36% and 74% , respectively, found SAP to be “very comfortable” or “relatively comfortable,” compared to 91% and 88%, respectively, for SVOP. The majority of subjects (71%) preferred SVOP, while 9% had no preference, and 20% preferred SAP (Fig. 6).

## Discussion

Our study showed that SVOP can be used to obtain threshold visual field sensitivity values in patients with glaucoma and produce maps of visual field defects with patterns exhibiting good agreement with SAP. This is an exciting finding as SVOP has the major advtange over SAP of being a totally objective test of visual function sindce it does not rely on a subjective patient response. Additionally, it negates the requirement of the patient to press a response button. Therefore, SVOP represents a potentially important new test for the evaluation of the visual field. An additional benefit is the finding that 71% of participants preferred SVOP to conventional SAP testing.

We used two versions of the SVOP software, with v2 found to be superior to v1, which is now retired. Compared to the gold standard of SAP, SVOP v2 correctly identified 42 of 43 abnormal visual fields, which equates to a sensitivity of 97.7%. Of 68 normal visual fields on SAP, 15 were identified as abnormal on SVOP v2. This indicated a specificity of 77.9% for SVOP compared to SAP SITA-fast; however, some of the normal SITA-fast tests might have been false-negatives. The study population for SVOP v2 testing included 70 eyes with glaucoma, of which 67 (95.7%) were identified as abnormal by SVOP and only 43 (61.4%) by SITA-fast (Table 2). Patients categorized as having glaucoma had an established clinical diagnosis by a glaucoma specialist based on the presence of glaucomatous optic disc changes and a visual field defect on SAP. Arguably, this is more meaningful than the results of an isolated SAP test, suggesting that many of the normal SITA-fast tests may have been false-negatives; for example, fellow eyes not yet manifesting glaucomatous field loss using an isolated SAP SITA-fast test. This hypothesis needs further testing as a limitation of the study was the lack of an independent reference standard for diagnosis.

We also found good agreement between patterns of field loss detected by SAP and SVOP. SVOP v2 produced visual field maps that were assigned to the same category as SAP for 76 of 111 (68%) eyes, with a further 20 (18%) differing by only one category. Only 15 of 111 tests (14%) differed by more than one category. For those cases where SAP and SVOP did not agree, the SVOP visual field tended to be graded with a higher number, normally indicating more severe visual field loss with 31 of 35 (89%) higher and 4 of 35 (11%) lower than SAP. The most frequent disagreement was normal (category 0) on SAP being classified as 1 or 3 by SVOP, and SAP category 3 or 4 being classified as 6 by SVOP. The tendancy for SVOP to produce results suggestive of a worse visual field than SAP is likely due to the difficulty of detecting small eye movements using the eye tracker, which may cause some inaccuracies in precise mapping of the central visual field in particular. Although the patient may perceieve and move fixation to a central stimulus, there may be instances where the eye tracker fails to detect this movement, resulting in an apparent scotoma that is not present on SAP. This finding also is supported by our separate analysis of agreement between SAP and SVOP threshold values, which found overall good agreement between these values, but poorer correlation between sensitivities in the central test locations.^17^ However, it is possible that some cases of apparantly worse visual fields on SVOP compared to SAP were due to earlier detection of damage using SVOP.

We found SVOP v2 to be superior than v1 and it is likely that further improvements are possible, particularly through software refinements and improvements in eye tracking technology. Towards the end of the study, an improved eye tracker became available (X2-60; Tobii Technology), with a higher sampling frequency (60 Hz compared to a variable 25–40 Hz used for the eye tracker used in v1 and v2). We subsequently evaluated the improved eye tracker (using software version 2) in 15 subjects (10 glaucoma patients, 5 healthy subjects), 11 of whom had experienced problems with the eye tracker and had incomplete tests during original testing. All 15 patients produced a complete test with 100% agreeing with the visual field defect classification from SAP. The results of testing in these 15 patients (available in full as Supplementary Material) suggest that further improvements in technology may reduce the number of incomplete tests and improve agreement with SAP; however, further studies are needed. We can speculate that improvements have been made to the hardware and firmware used by the eye tracker; however, these improvements are proprietary to the eye tracking company and it is not known exactly what factors contributed to the improved eye tracking other than an increased sampling rate.

Although disagreement may be reduced by improvements in hardware and software, some disagreement between SVOP and SAP may be expected due to the different methodologies to assess the visual field. SVOP uses a one-dimensional response (visual recognition) compared to the two-dimensional response used by SAP; that is, visual recognition and a following motor component (button press) and it is possible that this different methodology itself could have an influence in the measurement. Additionally, subjects were tested using the HFA “SITA Fast” thresholding algorithm, which is known to have larger test–retest variability than the “SITA Standard” algorithm.

SVOP has some additional limitations, especially that the current display screen is unable to display stimuli brighter than 14 dB and that the current testing algorithm takes signficantly longer than SAP SITA Fast.^17^ However, despite the longer test time, there was little difference in how the participants perceived the testing time, with most subjects scoring the time “just right” for both tests (Fig. 4). Patients and healthy subjects found SVOP to be easier and more comfortable to perform than SAP, a likely reason why the SVOP testing time did not “feel” unnecessarily long. It is possible that patients with glaucoma, who may be weary from repeated SAP tests, may have preferred SVOP due to its novelty; however, we also found that “perimetry naïve” healthy subjects preferred SVOP to SAP.

Other limitations included the inability to complete tests, usually due to problems with the eye tracker, which also may struggle to function in patients with high refractive error. However, we found the newer iteration of SVOP software, and the new eye tracker could overcome this problem. The use of a faster sampling eye tracker also opens the intriguing possibility of analyzing saccadic latency during an SVOP test, which may provide a novel method of assessing the impact of glaucoma on visual function. Previous studies have found patients with glaucoma to have delayed saccadic latency compared to healthy subjects.^6,9,10^ For example, Mazumdar et al.^6^ used eye movement perimetry to evaluate saccadic reaction times to stimuli presented at locations corresponding to the 54 test points of SAP.^6^ Patients with glaucoma had prolonged saccadic reaction times, with a trend towards increasing reaction times with increasing disease severity. Further work is needed; however, there may be the potential to quantify saccades to provide additional information to assist with diagnosis and monitoring.

In conclusion, we demonstrated an alternative method of measuring threshold visual fields that is repeatable and compares well with the current gold standard when considering the clinically relevant visual field pattern produced. It has advantages of being ergonomically preferred with no postural constraints, allows dynamic fixation, and uses a natural inherent reflex. We also have demonstrated that improvements can be made through iteration (in hardware and software). Further work is required to evaluate SVOP v3 on a larger cohort of subjects while also collecting useful normative threshold data and saccadic latency data in glaucoma patients and healthy subjects.

## Supplementary Material

Supplement 1

Supplement 2

Supplement 3

Supplement 4
