# Supplementary material for: Comparison of Threshold Saccadic Vector Optokinetic Perimetry (SVOP) and Standard Automated Perimetry (SAP) in Glaucoma. Part II: Patterns of Visual Field Loss and Acceptability
Source: Transl Vis Sci Technol. 2017 Sep 6;6(5):4. doi: 10.1167/tvst.6.5.4 (PMC5588911; doi:10.1167/tvst.6.5.4)
Supplement: Supplement 1 [file tvst-06-05-02_s01.pdf]

Supplementary Figure 1. Examples of SAP and SVOP threshold and grey-scale results obtained from patients with glaucoma included in the study. Upper and lower panels show the SAP and SVOP threshold sensitivities respectively.

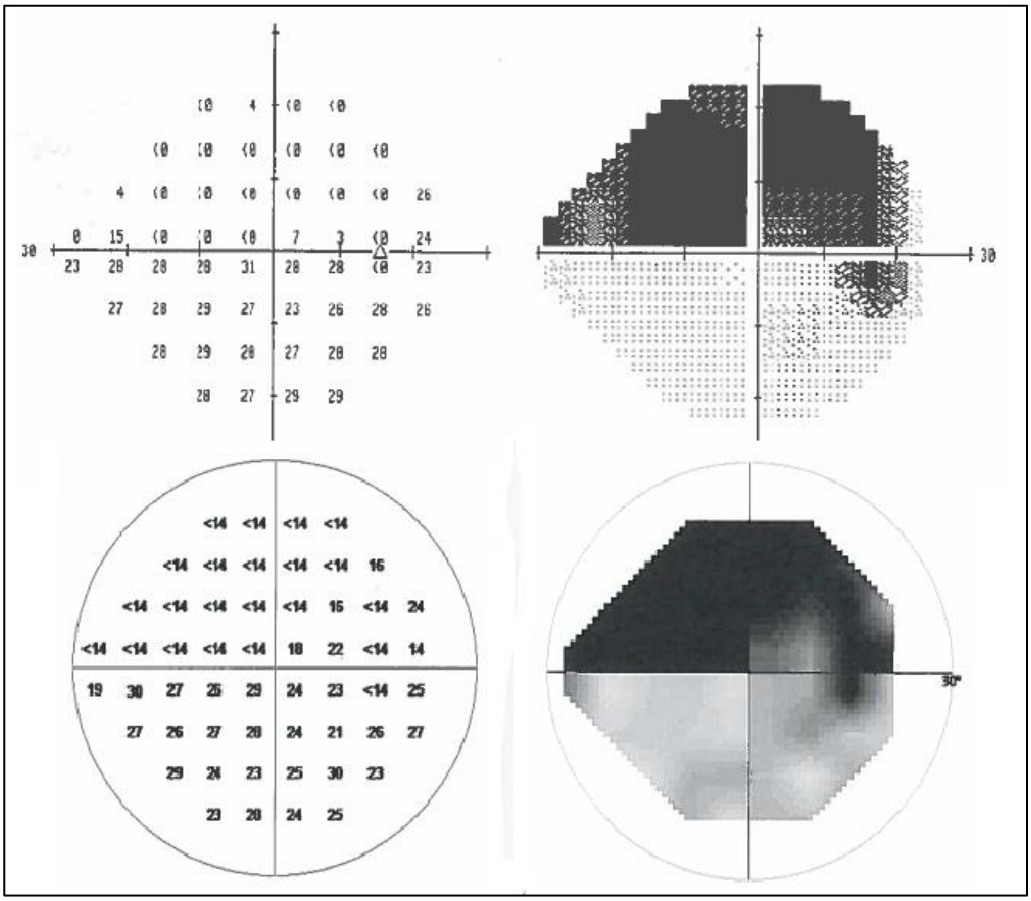

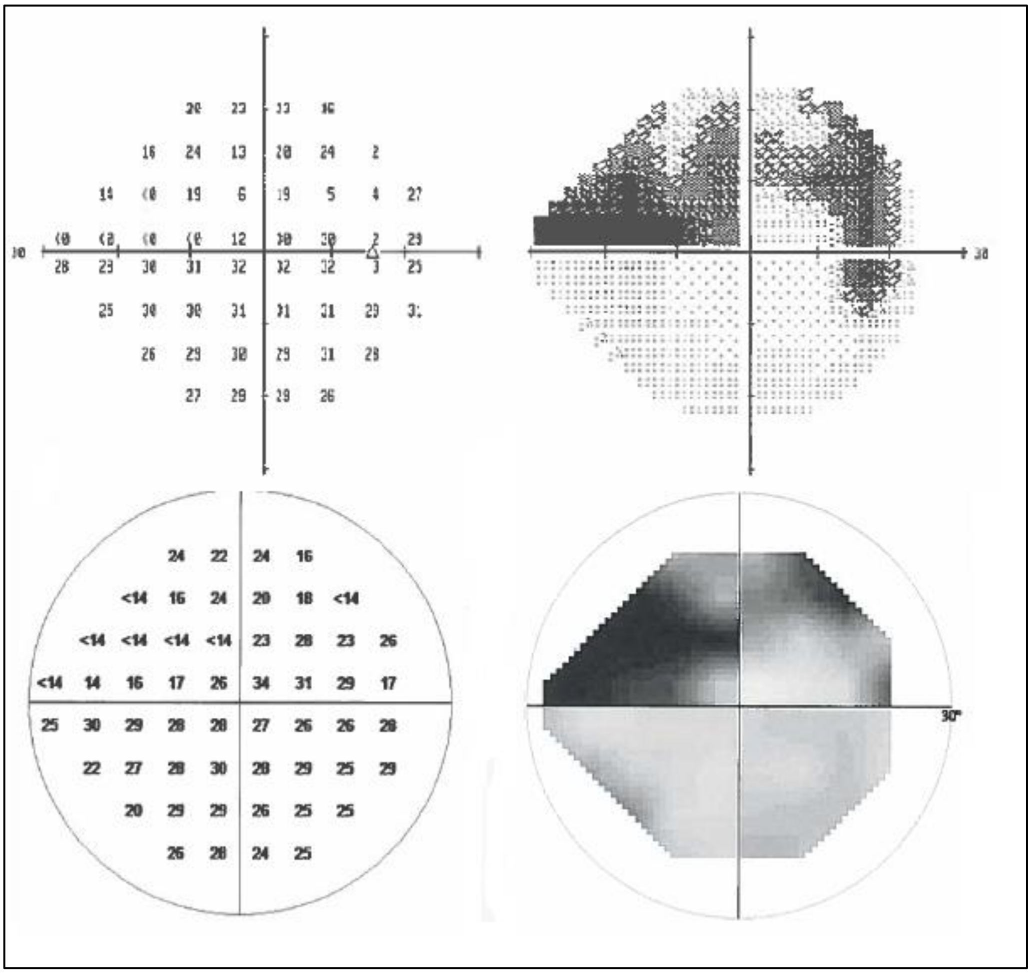

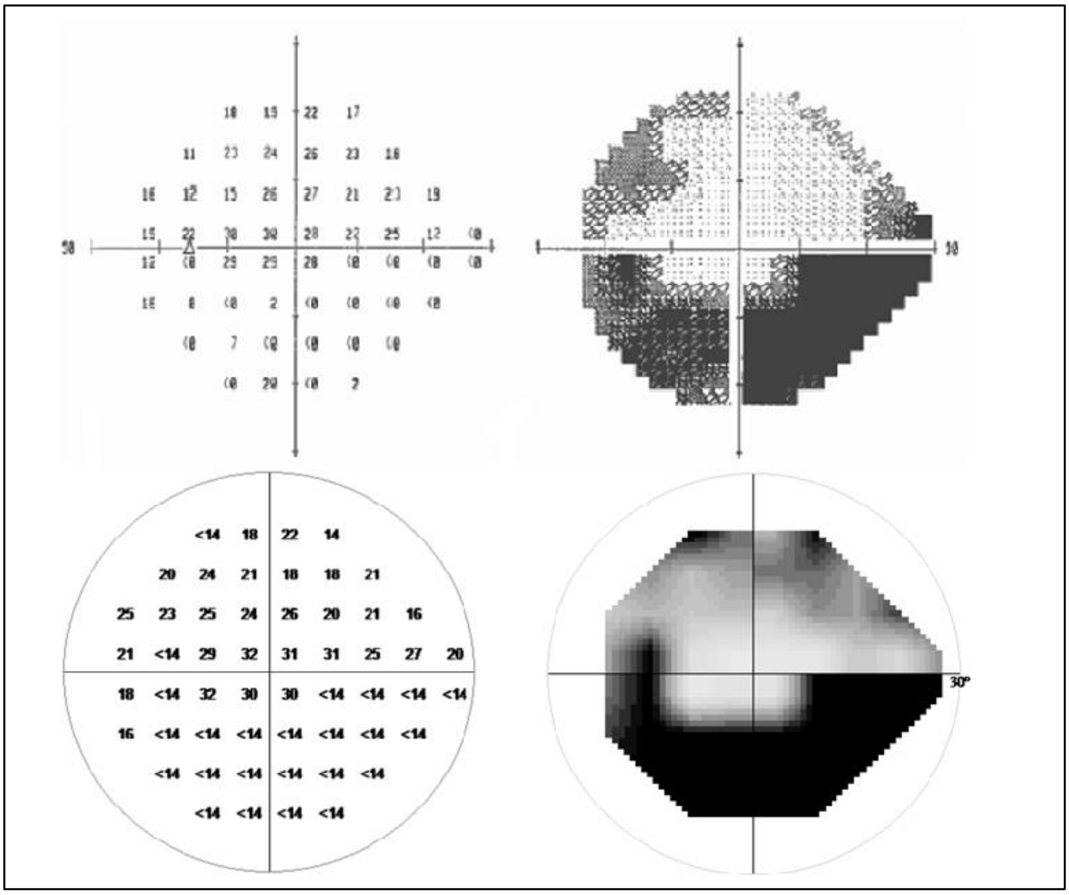

<14 18 22 14
20 24 21 18 18 21
25 23 25 24 26 20 21 16
21 <14 29 32 31 31 25 27 20
18 <14 32 30 30 <14 <14 <14 <14
16 <14 <14 <14 <14 <14 <14 <14
<14 <14 <14 <14 <14 <14
<14 <14 <14 <14
30°

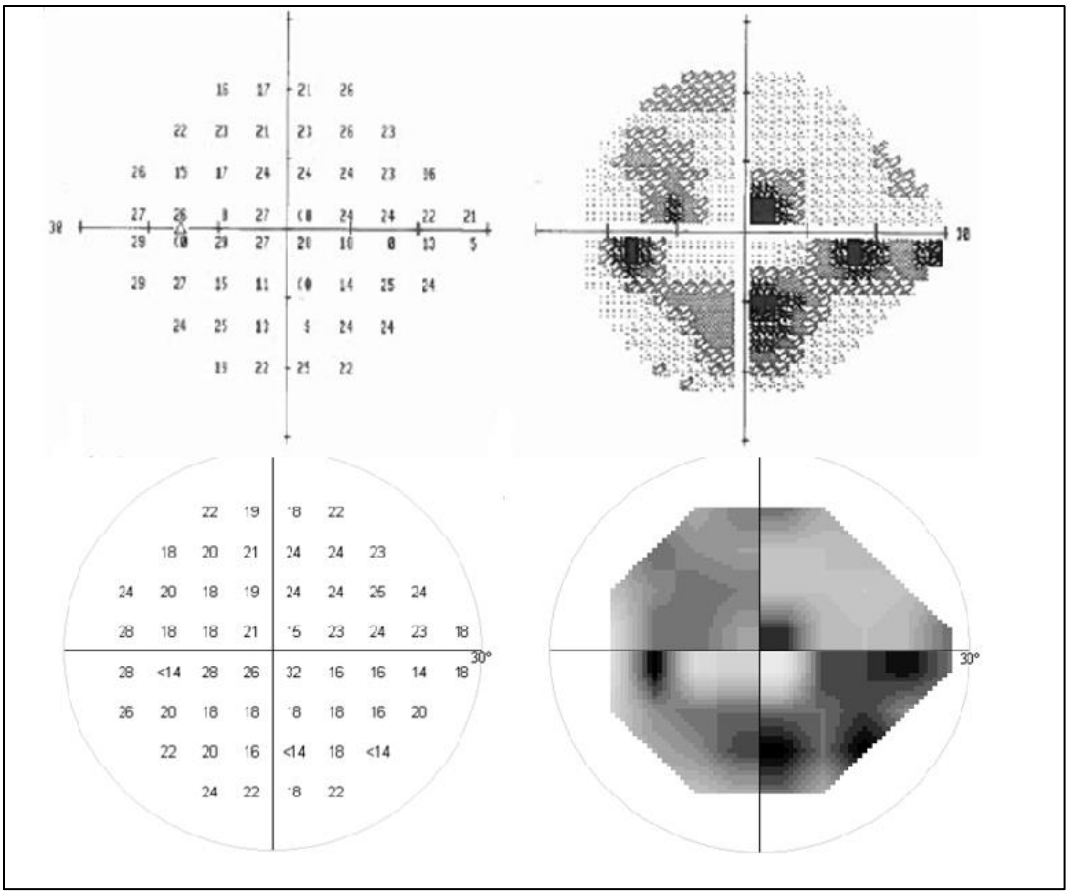

22 19 18 22
18 20 21 24 24 23
24 20 18 19 24 24 25 24
28 18 18 21 15 23 24 23 18
28 <14 28 26 32 16 16 14 18
26 20 18 18 18 18 16 20
22 20 16 <14 18 <14
24 22 18 22
30°
30°
