# Supplementary material for: Comparison of Threshold Saccadic Vector Optokinetic Perimetry (SVOP) and Standard Automated Perimetry (SAP) in Glaucoma. Part II: Patterns of Visual Field Loss and Acceptability
Source: Transl Vis Sci Technol. 2017 Sep 6;6(5):4. doi: 10.1167/tvst.6.5.4 (PMC5588911; doi:10.1167/tvst.6.5.4)

Supplementary Figure 2. Example of SAP and SVOP threshold and grey-scale results obtained from a healthy subject included in the study. Upper and lower panels show the SAP and SVOP threshold sensitivities respectively.

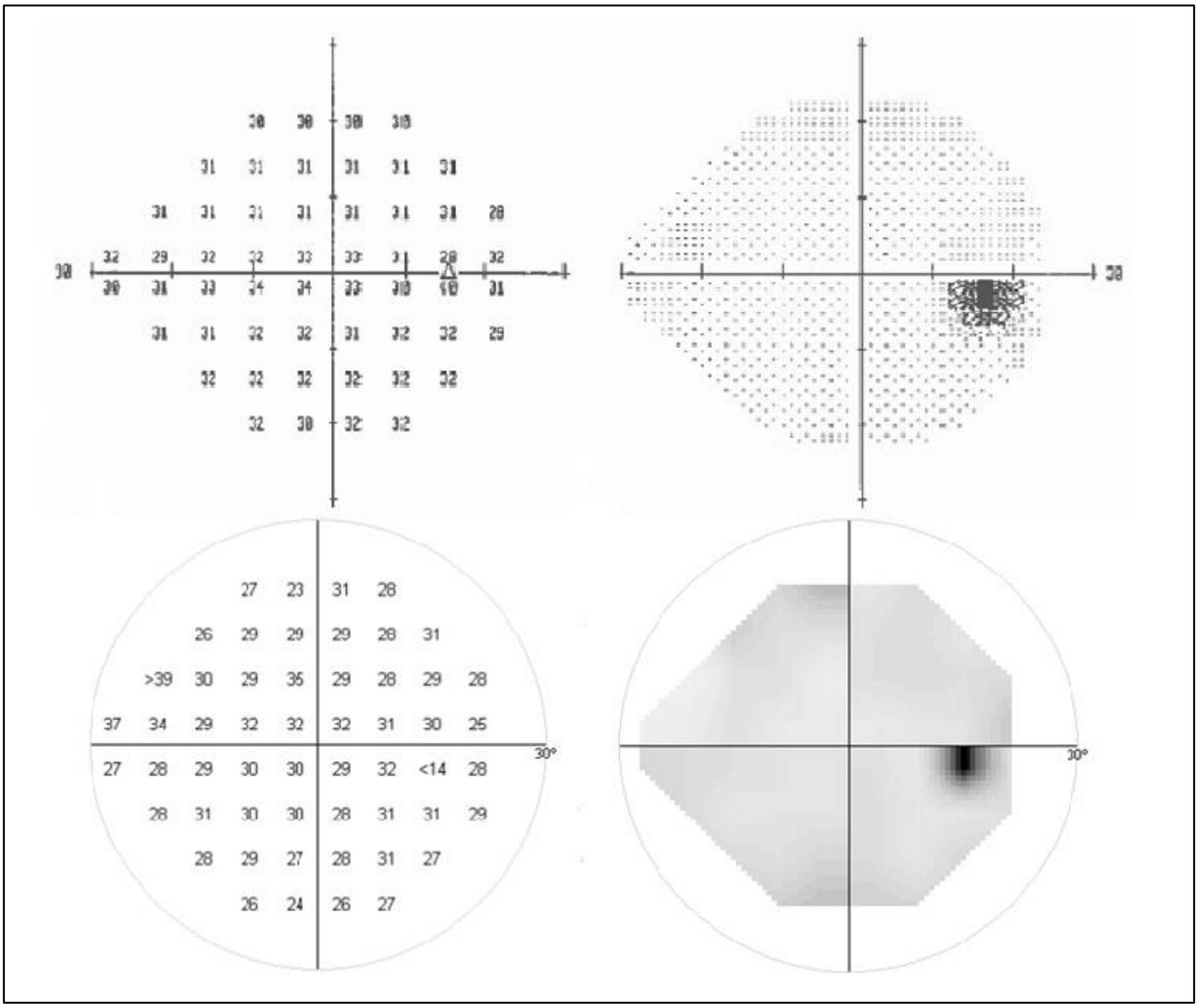

Supplement: Supplement 2 [file tvst-06-05-02_s02.pdf]
