# Supplementary material for: Comparison of Threshold Saccadic Vector Optokinetic Perimetry (SVOP) and Standard Automated Perimetry (SAP) in Glaucoma. Part II: Patterns of Visual Field Loss and Acceptability
Source: Transl Vis Sci Technol. 2017 Sep 6;6(5):4. doi: 10.1167/tvst.6.5.4 (PMC5588911; doi:10.1167/tvst.6.5.4)
Supplement: Supplement 3 [file tvst-06-05-02_s03.pdf]

Supplementary Table 1. Comparison of clinical visual field categories for SAP and SVOP v1 in healthy and glaucomatous subjects.

SVOP Version 1										
Group			SAP category							Total
			**0**	**1**	**2**	**3**	**4**	**5**	**6**	
**Glaucoma**	**SVOP category**	**0**	**23**	0	0	2	0	0	0	25
		**1**	5	**4**	0	0	0	0	0	9
		**2**	1	0	**1**	0	0	0	0	2
		**3**	5	6	3	**37**	0	0	0	51
		**4**	0	0	0	5	**1**	0	0	6
		**5**	0	0	0	0	0	**2**	0	2
		**6**	0	0	0	2	0	1	**0**	3
	**Total**		34	10	4	46	1	3	0	98
**Healthy**	**SVOP category**	**0**	24	0	1	0	0	0	0	25
		**1**	12	0	0	0	0	0	0	12
		**3**	7	0	0	0	0	0	0	7
	**Total**		43	0	1	0	0	0	0	44
