# Supplementary material for: Comparison of Threshold Saccadic Vector Optokinetic Perimetry (SVOP) and Standard Automated Perimetry (SAP) in Glaucoma. Part II: Patterns of Visual Field Loss and Acceptability
Source: Transl Vis Sci Technol. 2017 Sep 6;6(5):4. doi: 10.1167/tvst.6.5.4 (PMC5588911; doi:10.1167/tvst.6.5.4)
Supplement: Supplement 4 [file tvst-06-05-02_s04.pdf]

Supplementary Table 2. Results of testing with the new eye tracker (SVOP v3) compared to SVOP v2 and SAP.

Glaucoma (G) or healthy subject (H)	Visual field defect grading		
	SVOP v2	SVOP v3	SAP
G	3	4	4
G	2	2	2
G	Incomplete	2	2
G	3	3	3
G	3	3	3
G	Incomplete	0	0
G	2	2	2
G	0	0	0
G	2	3	3
G	Incomplete	3	3
H	0	0	0
H	Incomplete	0	0
H	Incomplete	0	0
H	0	0	0
H	0	0	0
